# Long-Term Macroscopic Performance, Hydration Characteristics, and Residual Hydration Capacity of Dam Concrete After More than 60 Years of Service

**DOI:** 10.3390/ma19194250

**Published:** 2026-10-08

**Authors:** Wenguang Jiang, Shimei Wang, Yan Shi, Yun Dong, Shihua Zhou, Jun Chen, Liang Zhang, Jiazheng Li, Ya Cao

**Affiliations:** 1 Institute of Materials and Structure, Changjiang River Scientific Research Institute, Wuhan 430010, China; 2 Key Laboratory of Changjiang River of Ministry of Water Resources, Wuhan 430010, China; 3 College of Mechanics and Materials, Hohai University, Nanjing 210098, China

**Keywords:** dam concrete, long-term service, macroscopic performance, hydration characteristics, residual hydration capacity

## Abstract

To characterize the material state and residual reactivity of long-service dam concrete, concrete cores extracted from the Lushui Reservoir Dam after more than 60 years of service were investigated. Their long-term macroscopic properties were evaluated, and the hydration products and microstructural features of the aged binder were characterized using X-ray diffraction, thermogravimetric analysis, scanning electron microscopy/energy-dispersive spectroscopy, and mercury intrusion porosimetry. The residual hydration capacity of the aged binder was further assessed through hydration heat and mortar strength tests. The results showed that the compressive strength of the concrete cores was mainly distributed within the range of 20–40 MPa, while the splitting tensile strength was mainly distributed within the range of 1–3 MPa, indicating that the concrete still retained relatively good basic mechanical properties after more than 60 years of service. The resistance to chloride ion penetration was generally favorable, whereas the frost resistance was relatively poor. The aged binder still contained identifiable Ca(OH)_2_, AFt, C-S-H, and CaCO_3_, and the local binder skeleton and aggregate–paste interface remained relatively stable. The residual hydration capacity of the aged binder was approximately 14–17%, indicating that the binder system had not become completely inert and still retained measurable residual reactivity. These findings provide a useful reference for evaluating the material state of long-service hydraulic concrete.

## 1. Introduction

Concrete is one of the most important construction materials in dam engineering, and its long-term service condition is directly related to structural safety, operational stability, and service-life assessment [1,2,3]. As many hydraulic and hydropower projects are progressively entering the long-term operation stage, identifying the material state, performance evolution, and potential durability risks of dam concrete based on real service samples has become an important scientific issue and engineering need in the maintenance and safety evaluation of existing structures [4,5,6]. Engineering practice has shown that, during long-term service, concrete dams may be affected by environmental actions, material aging, and limitations associated with early construction techniques, which can lead to deterioration in strength, impermeability, and frost resistance [7,8,9]. Therefore, investigating the material state of long-service dam concrete is of considerable practical significance [10,11].

Considerable research has been conducted on the performance evolution of long-age concrete [12,13,14]. Previous studies have shown that the mechanical behavior of long-age concrete does not follow a simple monotonic trend, but is strongly dependent on binder composition, curing conditions, and service environment [15,16,17]. Under favorable conditions, the compressive strength of concrete may continue to increase over many years, whereas under adverse conditions, its material properties may gradually deteriorate [18,19,20]. Studies on dam concrete cores have similarly indicated that concrete from some structures can still maintain relatively good mechanical properties after years or even decades of service, and may even exhibit continued strength development at later ages [21,22,23]. These findings suggest that the macroscopic performance evolution of long-service dam concrete is highly dependent on environmental and material factors, and cannot be simply generalized as either age-related deterioration or age-related improvement [24].

In addition to macroscopic properties, the long-term hydration state and microstructural evolution of the binder are also fundamental to the service behavior of concrete [25,26]. Previous studies have indicated that the later-stage hydration of cement-based materials is restricted by space availability [27,28]. However, under the relatively closed and stable internal environment of a dam, concrete may still undergo a certain degree of hydration even at very late ages [29,30]. Meanwhile, factors such as the state of hydration products, the characteristics of the interfacial transition zone, and the pore structure distribution may significantly influence macroscopic properties, including compressive strength, impermeability, and frost resistance [31,32]. In contrast, studies on the residual hydration capacity of binders in ultra-long-service dam concrete remain limited, particularly those based on real engineering cores and integrating macroscopic performance with microstructural characteristics [33,34,35]. Although a few studies have suggested that dam binders may still retain some reactivity after many years of hydration, this issue remains insufficiently understood for concrete with much longer service durations.

The Lushui Reservoir project has been in operation for more than 60 years. Part of its main dam was constructed as a gravity dam assembled using precast concrete blocks, providing a valuable opportunity to investigate the material state of long-service dam concrete. In this study, concrete cores extracted from the Lushui Reservoir Dam after more than 60 years of service were used as research objects. Their long-term macroscopic properties were systematically evaluated, and the hydration products and microstructural features of the aged binder were characterized. In addition, the residual hydration capacity of the aged binder was quantitatively assessed using hydration heat and mortar strength tests. This study aims to elucidate the material state and residual reactivity of long-service dam concrete and to provide a reference for the state identification and material-level evaluation of long-age hydraulic concrete.

## 2. Materials and Methods

### 2.1. Engineering Background and Core Sampling

Construction of the Lushui Reservoir project commenced in October 1958, and the hydraulic complex was completed in 1976. Including the periods of construction, experimental investigation, long-term operation, and management, the project has been in service for more than 60 years. The hydraulic complex consists of the main dam, 12 auxiliary dams, spillway structures, a powerhouse, a switch station, north and south irrigation canal intakes, and a simple vertical boat lift for dry transport. Among these structures, the main dam and the No. 3 auxiliary dam are gravity dams assembled from precast concrete blocks. Of the 14 monoliths of the main dam, 12 were constructed using the precast assembly method, except for monoliths 13 and 14. The precast blocks were mainly connected by masonry-type jointing. The horizontal joints were filled with cement mortar, with a compacted thickness of approximately 2 cm, whereas the vertical joints were filled with concrete, with a joint width of approximately 15–20 cm. In some local dam sections, a grouted bonding method using preplaced aggregate in the vertical joints was also adopted.

**Figure 1:**
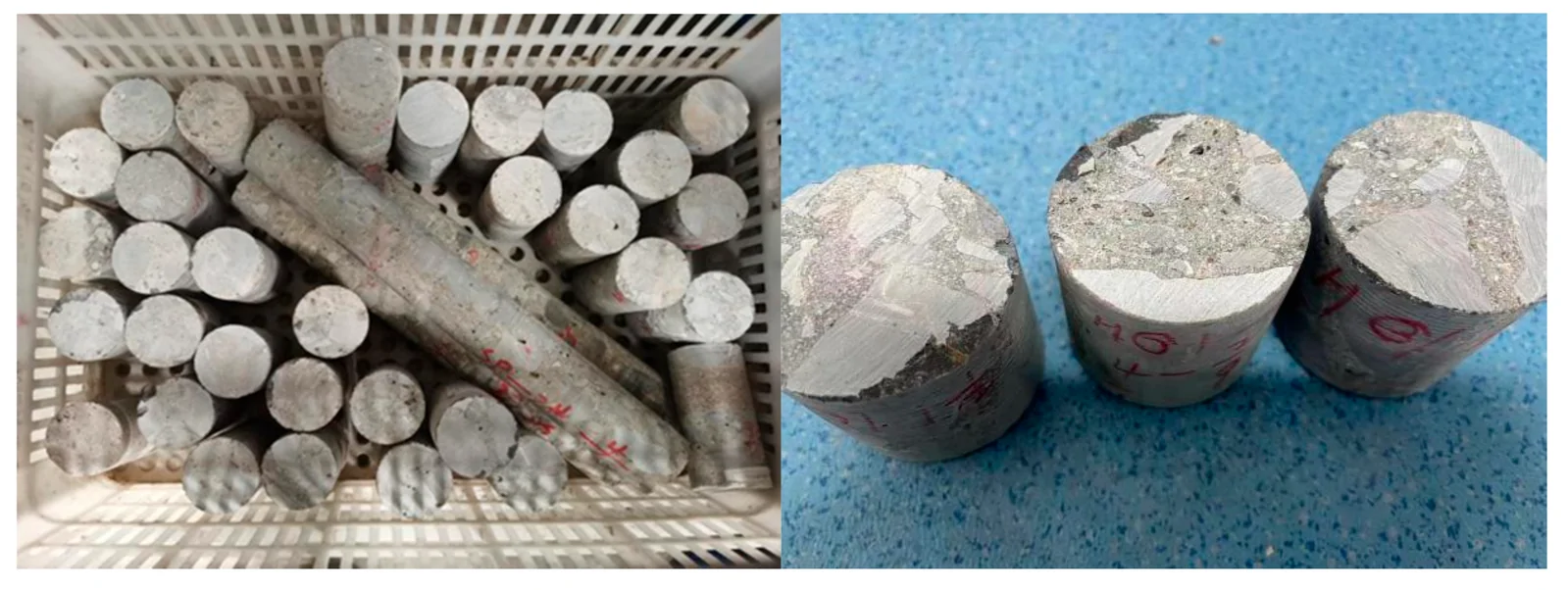
Concrete core specimens extracted from the Lushui Dam.

The specimens used in this study were mainly obtained from monolith No. 3 of the main dam. Concrete cores were drilled in both horizontal and vertical orientations, and photographs of the cores are shown in Figure 1. According to the actual core length and the presence of defects, the cores were screened and processed to satisfy the dimensional requirements of different tests. It should be noted that the specimens used for mechanical testing did not contain horizontal mortar joints or vertical concrete joints. Therefore, the present study mainly reflects the long-term material state of the precast concrete blocks themselves, rather than the overall response of the block–joint composite system.

### 2.2. Testing of Long-Term Macroscopic Properties

#### 2.2.1. Basic Physical and Mechanical Tests

The basic physical and mechanical tests included bulk density, compressive strength, splitting tensile strength, and compressive elastic modulus. Bulk density was determined in accordance with SL/T 352-2020 [36], Test Code for Hydraulic Concrete. A density of 2400 kg/m^3^ was used as the reference value for ordinary concrete, and the ratio of the measured bulk density to this reference value was used to evaluate the density state of the long-service cores.

Compressive strength, splitting tensile strength, and compressive elastic modulus were also measured in accordance with SL/T 352-2020. Since the tested core specimens did not contain horizontal mortar joints or vertical concrete joints, the measured results mainly represent the long-term mechanical state of the precast concrete blocks themselves. In addition, because the drilling paths may have crossed different casting layers and the local heterogeneity of the samples was relatively pronounced, the results were discussed based on individual core values rather than simply averaging all specimens.

The specimens used for bulk density testing had diameters of approximately 54.3–54.4 mm and heights of approximately 54–56 mm. The specimens used for compressive strength and splitting tensile strength tests had diameters of approximately 54.3–54.4 mm and heights of approximately 54–56 mm, whereas those used for elastic modulus measurement had diameters of approximately 54.3–54.4 mm and heights of approximately 108–110 mm. Owing to the limited number and size of the cores, only one axial tensile specimen was obtained; therefore, this index was not included in the main discussion of this paper.

#### 2.2.2. Frost Resistance Test

The frost resistance of the core specimens was evaluated in accordance with SL/T 352-2020. Because the core specimens were not standard frost-resistance specimens, mass loss and relative dynamic modulus could not be used simultaneously for a comprehensive assessment. Therefore, frost resistance in this study was mainly characterized by the mass loss during freeze–thaw cycling and the failure condition of the specimens.

Five groups of core specimens were tested for frost resistance, with specimen dimensions of approximately φ54.3–54.4 mm × 162.8–163.2 mm. Considering the limitations associated with coring, specimen processing, and specimen size, the present results are more suitable for identifying the relative freeze–thaw resistance of the long-service cores than for direct comparison with frost-resistance grades determined using standard large-size specimens.

#### 2.2.3. Chloride Ion Penetration Test

**Figure 2:**
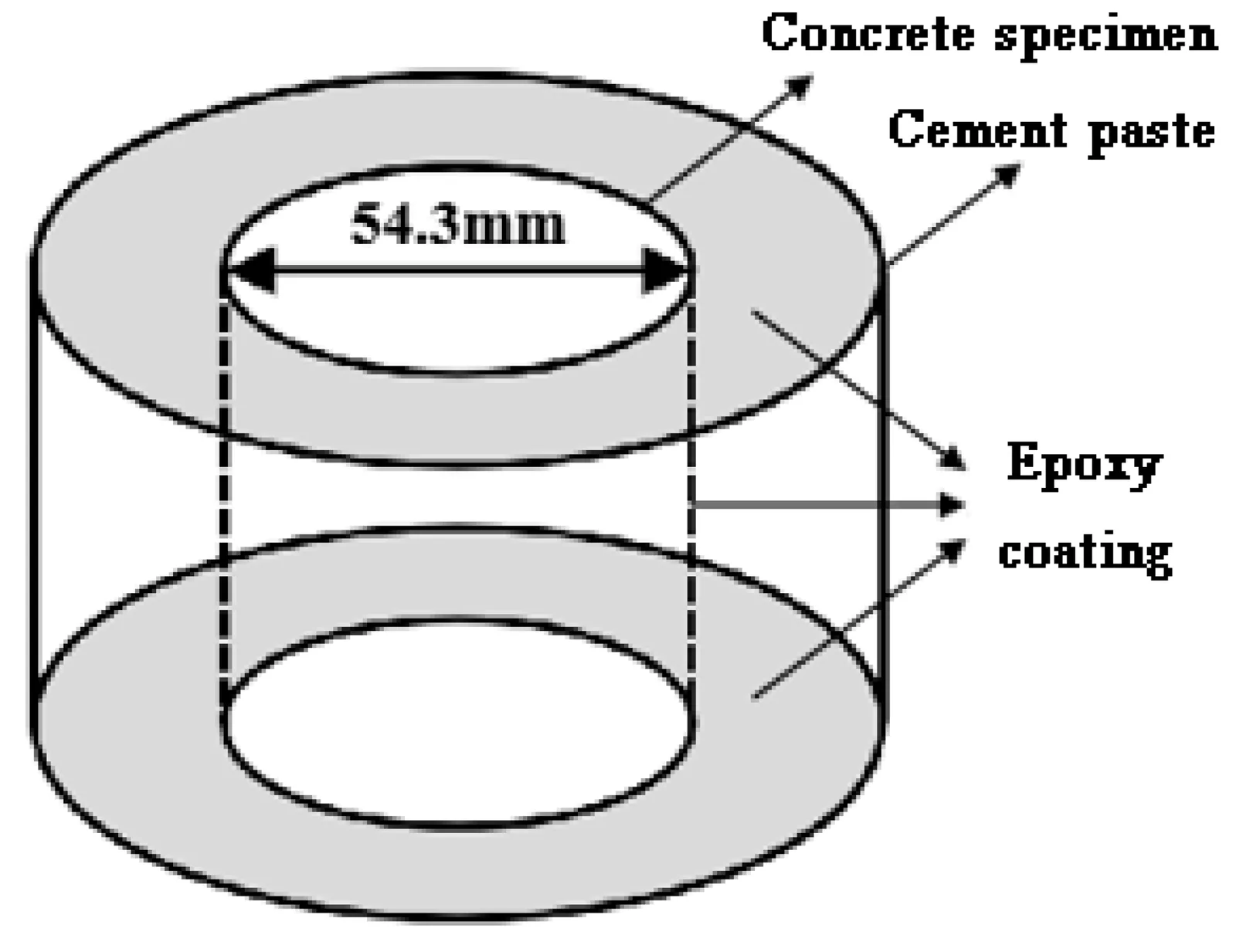
Schematic illustration of the modified specimen used for chloride ion penetration testing.

The resistance to chloride ion penetration was evaluated using the electric flux method specified in SL/T 352-2020. Because the original cores had diameters of only 54.3–54.4 mm and therefore did not meet the dimensional requirements for standard test specimens, the cores were first processed into cylinders of the required height, and their curved outer surfaces were coated with epoxy resin. After curing for 7 d, each core was placed at the center of a mold with a diameter of 100 mm and a height of 50 mm, and fresh paste was cast around it. After curing for 28 d, the non-core areas on both end surfaces of the specimen were coated again with epoxy resin and cured for 7 d before testing. A schematic illustration of the modified specimen configuration is shown in Figure 2.

Based on the measured total electric charge passing through the core, an equivalent electric flux corresponding to a standard specimen with a diameter of 95 mm was calculated according to the proportional relationship between electric flux and cross-sectional area. The chloride ion penetration level was evaluated in accordance with JGJ/T 193-2009 [37], Standard for Inspection and Assessment of Concrete Durability. It should be noted that the obtained value represents the relative impermeability level of the small-sized core under the modified specimen condition, rather than a direct measurement obtained from a standard full-size original specimen.

### 2.3. Characterization of Hydration Products and Microstructure

#### 2.3.1. XRD Test

After crushing the dam concrete cores, hardened binder fragments were manually selected and dried in a vacuum oven at 60 °C for 24 h. The dried material was then ground in an agate mortar, with care taken to minimize overgrinding of entrained sand particles, and finally passed through a 200-mesh sieve. X-ray diffraction (XRD) analysis was performed using an Empyrean X-ray diffractometer over a scanning range of 5–70° at a scanning rate of 5°/min to identify the crystalline phases in the aged binder.

#### 2.3.2. TG Test

The samples used for thermogravimetric (TG) analysis were the same as those used for XRD analysis. Thermal analysis was performed using an STA449F3 simultaneous thermal analyzer (NETZSCH-Gerätebau GmbH, Selb, Germany) over a temperature range from room temperature to 1000 °C, at a heating rate of 10 °C/min under a nitrogen atmosphere. This test was conducted to characterize the thermal decomposition behavior and major phase composition of the aged binder.

#### 2.3.3. SEM and EDS Test

Hardened binder fragments were manually selected from the crushed core material and dried in a vacuum oven at 60 °C for 24 h before observation. Scanning electron microscopy (SEM) analysis was conducted using a JSM-6610LV scanning electron microscope (JEOL Ltd., Akishima, Tokyo, Japan) to examine the microstructural morphology of the aged binder. To investigate the characteristics of the aggregate–paste interfacial transition zone, selected aggregate–binder contact regions were observed by SEM, and energy-dispersive spectroscopy (EDS) was further used to analyze the elemental distribution across the interface.

#### 2.3.4. MIP Test

Mercury intrusion porosimetry (MIP) was conducted using an AutoPore IV 9500 porosimeter (Micromeritics Instrument Corporation, Norcross, GA, USA). The sample preparation procedure was the same as that used for SEM analysis; that is, hardened binder fragments were selected from the crushed cores and dried in a vacuum oven at 60 °C for 24 h before testing. Porosity, pore volume, average pore diameter, most probable pore diameter, and pore size distribution were obtained to characterize the pore structure of the aged binder. In this study, pores were classified into three categories according to pore diameter: pores smaller than 50 nm were defined as harmless pores, those between 50 and 100 nm as less harmful pores, and those larger than 100 nm as harmful pores.

### 2.4. Evaluation of Residual Hydration Capacity

To evaluate the residual hydration capacity of the aged binder, hardened binder fragments were separated from the dam concrete cores, dried in a vacuum oven at 60 °C for 24 h, finely ground, and passed through a 200-mesh sieve. The resulting aged binder powder was used as a partial replacement for fresh cement in paste and mortar mixtures for hydration heat and strength tests. Meanwhile, quartz sand was also finely ground and passed through a 200-mesh sieve, and the resulting quartz powder was used as an inert reference material to partially replace cement. A pure cement system was used as the fully active reference. By comparing the aged-binder replacement system with the inert quartz replacement system and the pure cement system, the relative residual activity of the aged binder in fresh mixtures was assessed.

The fresh cement used in this study was P.MH 42.5 moderate-heat Portland cement produced by Jiahua Special Cement Co., Ltd. (Leshan, Sichuan, China). Its main physical properties and chemical composition are listed in Table 1 and Table 2. All indices satisfied the technical requirements of GB/T 200-2017 [38], Moderate Heat Portland Cement and Low Heat Portland Cement.

For the aged dam concrete, the phase composition of the binder was characterized by XRD and TG analyses (Section 2.3 and Section 3.2), which identified CaCO_3_, Ca(OH)_2_, and AFt as the main phases. The elemental composition of the aged dam concrete was not determined because it is dominated by the chemically inert siliceous aggregate, which would mask the binder signal and provide limited information on the hydration state relevant to residual hydration capacity.

#### 2.4.1. Evaluation Method Based on Hydration Heat

Three paste mixtures were designed for the hydration heat test: pure cement paste (CP), paste with 30% of the cement replaced by ground quartz powder (SP), and paste with 30% of the cement replaced by aged binder powder (LP). All mixtures had a water-to-binder ratio of 0.5, and the mix proportions are listed in Table 3.

The hydration heat evolution and cumulative heat release within 72 h were measured using a microcalorimeter. Since the pure cement system can be regarded as the 100% active reference and the quartz replacement system as the inert reference, the position of the LP system relative to these two limits was used to characterize the residual hydration capacity of the aged binder.

The residual hydration capacity calculated from hydration heat was determined using Equation (1):

(1) $$K_{h}=\frac{h_{\mathrm{LP}}-h_{\mathrm{SP}}}{h_{\mathrm{CP}}-h_{\mathrm{SP}}}\times 100\%$$

where *K_h_* is the residual hydration capacity based on hydration heat, and *h_CP_*, *h_LP_*, and *h_SP_* are the 72 h cumulative heat releases of the CP, LP, and SP systems, respectively.

#### 2.4.2. Evaluation Method Based on Mortar Strength

Three mortar mixtures were also prepared: pure cement mortar (CM), mortar with 30% of the cement replaced by ground quartz powder (SM), and mortar with 30% of the cement replaced by aged binder powder (LM). All mortars had a water-to-binder ratio of 0.5 and a binder-to-sand ratio of 1:3. The mix proportions are listed in Table 4.

Mortar specimens were prepared, cured, and tested for compressive and flexural strengths in accordance with GB/T 17671-2021 [39], Test Method of Cement Mortar Strength (ISO Method).

Because mortar strength still increases rapidly before 28 d, the residual hydration capacity of the aged binder was calculated using the compressive strengths at 28 and 90 d to improve the stability of the evaluation. The calculation is given in Equation (2):

(2) $$K_{s}=\frac{s_{\mathrm{LP}}-s_{\mathrm{SP}}}{s_{\mathrm{CP}}-s_{\mathrm{SP}}}\times 100\%$$

where *K_s_* is the residual hydration capacity based on mortar compressive strength, and *s_CP_*, *s_LP_*, and *s_SP_* are the compressive strengths of the CM, LM, and SM systems, respectively.

It should be noted that the “residual hydration capacity” defined in this study is a relative index derived from the overall response of the replacement systems. This index may include not only the direct contribution of components in the aged binder that still retain reaction potential, but also combined effects such as ion release, nucleation promotion, and microfilling. Therefore, it is more appropriate to interpret this value as the overall residual activity of the aged binder in fresh mixtures, rather than as a direct measure of the absolute amount of unhydrated phases or the activity of any single reactive component.

## 3. Results and Discussions

### 3.1. Macroscopic Performance After More than 60 Years of Service

#### 3.1.1. Apparent Density Characteristics

**Figure 3:**
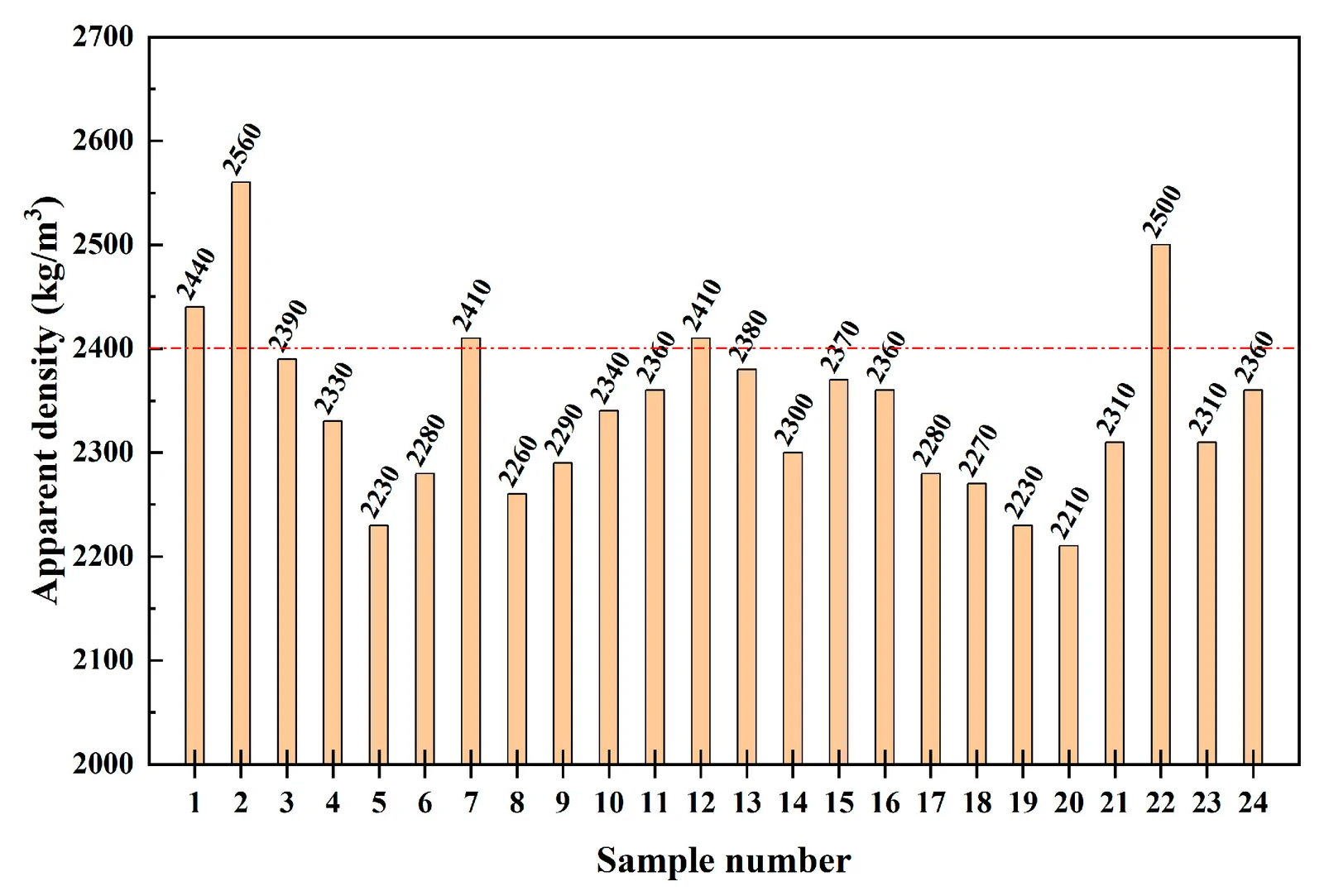
Apparent density of the dam concrete cores.

The apparent density results of the dam concrete cores are shown in Figure 3. The apparent density of the different samples ranged from 2210 to 2560 kg/m^3^. Relative to the reference value of 2400 kg/m^3^ for ordinary concrete, the corresponding ratios ranged from 92.1% to 106.7%, and approximately three-quarters of the specimens were within the range of 95–105%. These results indicate that the apparent density of the core samples generally fluctuated within a normal range, without abnormally low values or pronounced dispersion.

Apparent density can, to some extent, reflect whether the overall material state of concrete exhibits obvious loosening or abnormal pore development. The present results suggest that, after more than 60 years of service, the precast concrete blocks of the Lushui Dam still maintained a relatively stable overall physical state and showed no clear signs of global loosening. The relatively high apparent density observed in several individual samples may be attributed to local aggregate concentration or material heterogeneity associated with early construction conditions. It should be noted that apparent density mainly reflects the overall physical state of the material and is relatively insensitive to local microcracks and fine pore structure. Therefore, in this study, its significance lies primarily in providing a basic reference for the subsequent analyses of mechanical properties and microstructure.

#### 3.1.2. Mechanical Properties: Compressive Strength and Splitting Tensile Strength

**Figure 4:**
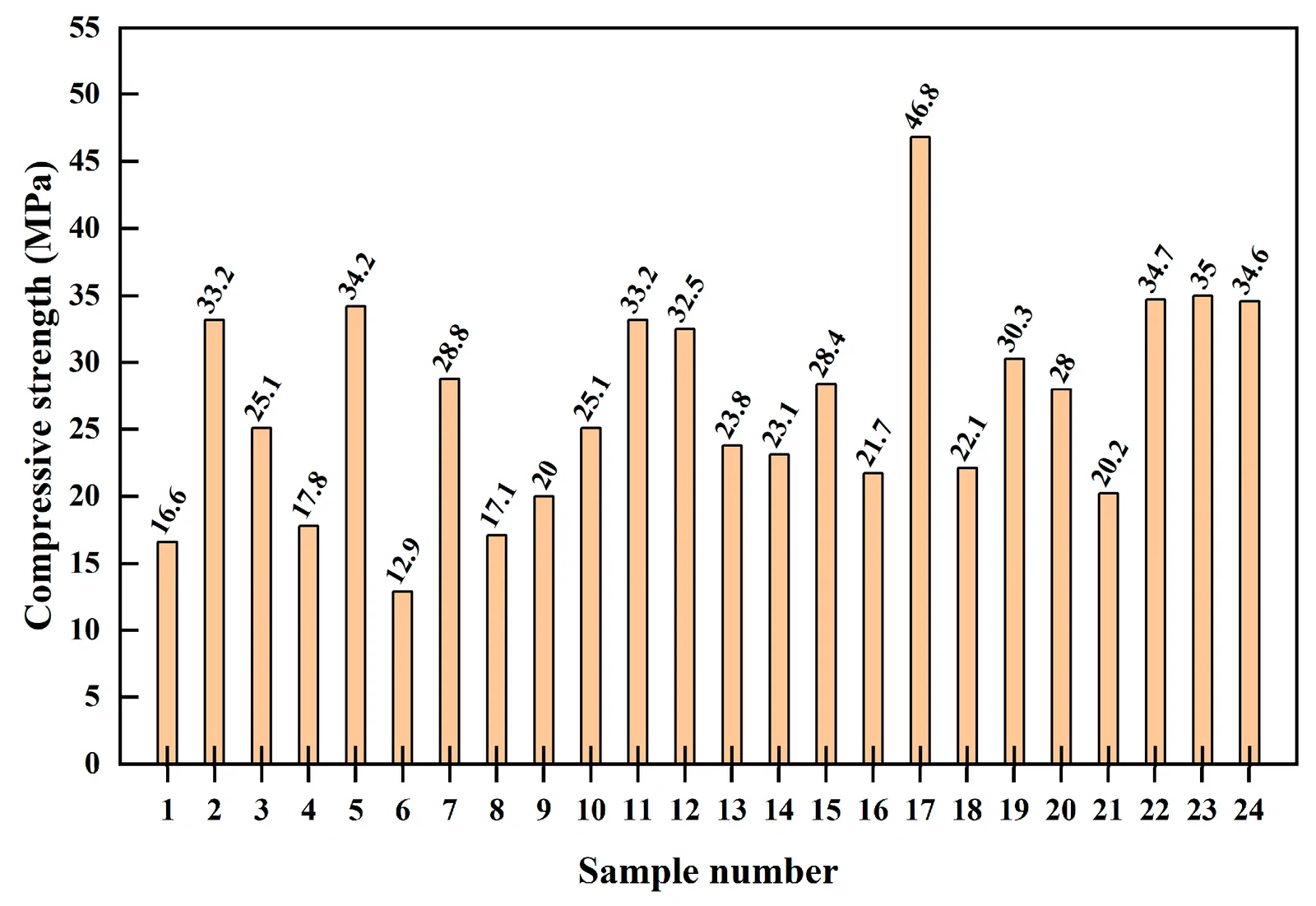
Compressive strength of the dam concrete cores.

The compressive strength results of the dam concrete cores are shown in Figure 4. The compressive strength of the different samples ranged from 12.9 to 46.8 MPa. The proportions of specimens within the ranges of 10.0–20.0 MPa, 20.0–30.0 MPa, 30.0–40.0 MPa, and >40.0 MPa were 20.8%, 41.7%, 33.3%, and 4.2%, respectively. In addition, the compressive strength of these cores had a median of 26.6 MPa and a mean of 26.9 MPa, with a relatively large standard deviation of 7.9 MPa, indicating considerable dispersion of the compressive strength. Overall, the compressive strength test results indicate that these long-service cores still retained relatively stable compressive load-bearing capacity after more than 60 years of service. Considering that the original design grade of the precast blocks was 75#, approximately equivalent to C5.5, the measured compressive strengths were substantially higher than the originally documented design level. This suggests that, at least in the sampled region investigated in this study, the precast concrete blocks did not undergo overall strength deterioration during long-term service.

**Figure 5:**
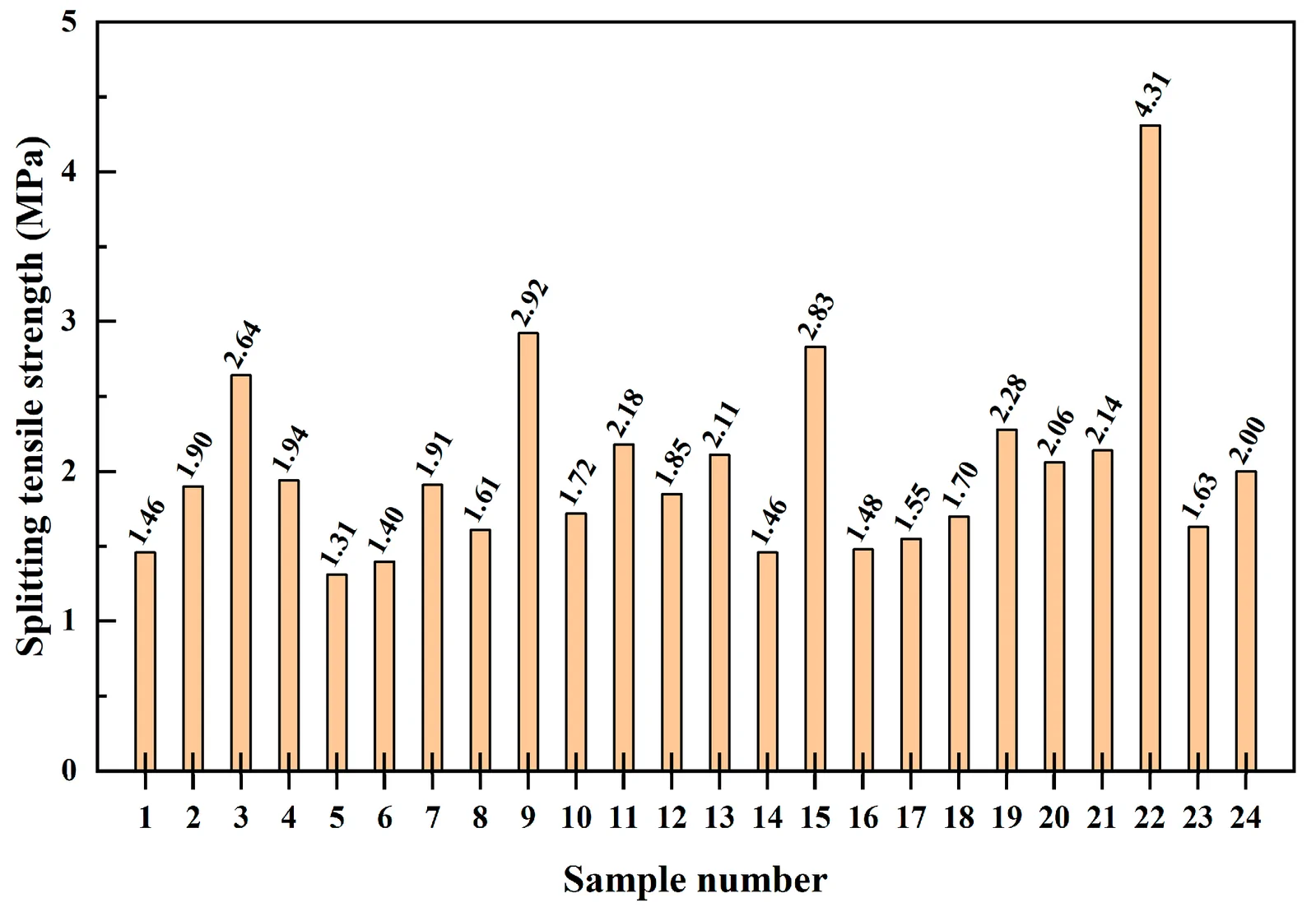
Splitting tensile strength of the dam concrete cores.

The splitting tensile strength results are shown in Figure 5. The splitting tensile strength ranged from 1.31 to 4.31 MPa, with 62.5% of the specimens distributed within the range of 1.00–2.00 MPa, 33.3% within the range of 2.00–3.00 MPa, and only 4.2% exceeding 3.00 MPa. In addition, the splitting tensile strength of these cores had a median of 1.90 MPa and a mean of 2.02 MPa, with a standard deviation of 0.65 MPa. Compared with compressive strength, the splitting tensile strength showed lower absolute values and a relatively more concentrated distribution, which is consistent with the inherent sensitivity of tensile behavior to internal defects in concrete. Nevertheless, no abnormal clustering of extremely low values was observed, indicating that the binder skeleton and aggregate–paste interface still maintained a certain degree of integrity after long-term service.

Taken together, the compressive and splitting tensile strength results indicate that the Lushui Dam cores still retained relatively good basic mechanical properties after more than 60 years of service. Previous studies have shown that, under relatively stable environmental conditions, the strength of long-age concrete may continue to develop, and some dam concrete cores may even exhibit further strength gain after years or decades of service. The present results are generally consistent with this understanding, suggesting that the mechanical performance evolution of long-service dam concrete is not necessarily dominated by degradation, but is closely related to the service environment, the persistence of hydration, and the internal structural state of the material.

On the other hand, the wide range of compressive strength values, from 12.9 to 46.8 MPa, also indicates considerable variability among individual samples. Considering that the cores were extracted from Monolith No. 3 of the main dam, that the drilling paths may have crossed different placement layers, and that the distribution of aggregates, local compactness, and internal defects may not have been identical owing to early construction conditions, such variability is considered reasonable. It should also be emphasized that the mechanical specimens did not contain horizontal mortar joints or vertical concrete joints; therefore, the results mainly reflect the long-term mechanical state of the precast concrete blocks themselves, rather than the overall structural response of the assembled dam system.

#### 3.1.3. Deformation-Related Property: Compressive Elastic Modulus

**Figure 6:**
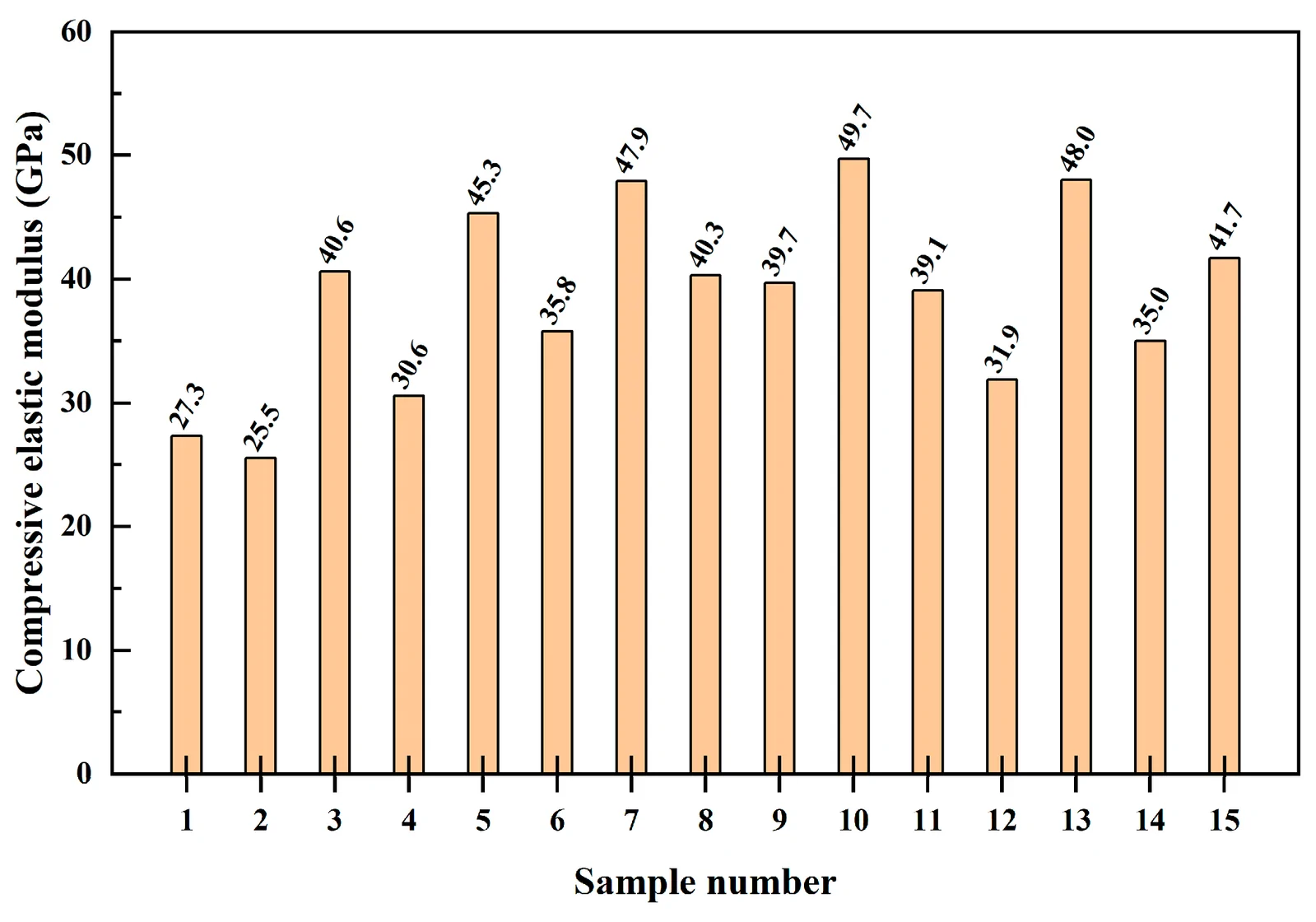
Compressive elastic modulus of the dam concrete cores.

The compressive elastic modulus results of the dam concrete cores are shown in Figure 6. The compressive elastic modulus of the different samples ranged from 25.5 to 49.7 GPa, showing a relatively wide distribution. Compared with the compressive strength, which was mainly concentrated in the range of 20–40 GPa, the elastic modulus exhibited more pronounced variability, indicating considerable differences in the deformation responses of individual cores under compressive loading.

The compressive elastic modulus is more sensitive than compressive strength to aggregate properties, aggregate volume fraction, aggregate distribution, the condition of the interfacial transition zone, and internal microcracks. Therefore, its variability can more effectively reflect the spatial heterogeneity of the material. The present results suggest that, although the long-service cores from the Lushui Dam still retained relatively good overall load-bearing capacity, differences remained among individual samples in terms of local skeleton integrity and deformation compatibility. Considering the early construction period of the project, as well as possible aggregate concentration or segregation during fabrication of the precast blocks, such heterogeneity is considered reasonable. This result also indicates that, even when the load-bearing performance of long-service dam concrete remains relatively stable, its internal mesostructural state may still vary from one location to another, providing a basis for the subsequent analysis of pore structure and interfacial characteristics.

#### 3.1.4. Durability-Related Properties: Frost Resistance and Chloride Ion Penetration Resistance

The frost resistance results of the dam concrete cores are listed in Table 5. Among the five tested specimens, four failed within 25 freeze–thaw cycles, while only one specimen survived 25 cycles with a mass loss of 0.83%, but still failed within 50 cycles. These results indicate that the frost resistance of the long-service cores was generally poor.

The chloride ion penetration resistance results are presented in Table 6. The equivalent electric flux values were 3980, 2820, 1620, 3240, and 3120 C, respectively. Four specimens were within the range of 2000–4000 C, corresponding to a moderate chloride ion permeability level, while one specimen was within the range of 1000–2000 C, corresponding to a low permeability level. This indicates that the chloride ion penetration resistance of the cores was generally favorable. It should be noted that the original mix composition of the precast blocks, including the possible use of mineral admixtures such as slag or fly ash, is no longer available after more than 60 years of service. Nevertheless, the present results were obtained directly from the extracted cores and reflect the integrated state of the concrete after long-term service; the material-level conclusion of generally favorable chloride ion penetration resistance is therefore independent of the unknown initial composition.

The favorable chloride ion penetration resistance of the cores can be partly attributed to the chloride binding capacity of the hydration products. Chloride ions that penetrate into the concrete can react with the AFm phases to form Friedel’s salt, as follows:

C_3_A + CaCl_2_ + 10H_2_O → C_3_A · CaCl_2_ · 10H_2_O (Friedel’s salt) (3)

C_3_A · CaSO_4_ · 12H_2_O (AFm) + 2NaCl → C_3_A · CaCl_2_ · 10H_2_O (Friedel’s salt) + Na_2_SO_4_ + 2H_2_O (4)

The formation of Friedel’s salt immobilizes a portion of the penetrated chloride ions and contributes to a denser pore structure, thereby reducing the effective chloride transport rate and supporting the favorable chloride ion penetration resistance observed for the cores.

Taken together, the frost resistance and chloride ion penetration results reveal a clear divergence in the durability-related performance of the long-service dam concrete. In contrast to the relatively good resistance to chloride ion penetration, the frost resistance was markedly weaker. This suggests that different durability indicators do not necessarily evolve synchronously during long-term service. Compared with chloride ion transport, freeze–thaw damage is more sensitive to local large pores, microcracks, and unfavorable pore-size distributions. Therefore, even when the material still exhibits relatively good resistance to ion transport, it may remain vulnerable to cyclic freeze–thaw action.

Considering the engineering background, this difference may be related to the relatively low historical design requirement for frost resistance in the Lushui region, as well as the cumulative mesoscopic damage that developed during long-term service. It should also be noted that the frost resistance specimens were non-standard specimens, and the chloride ion penetration values were obtained from modified specimens with cross-sectional conversion. Accordingly, the present results are more appropriate for identifying the relative durability state of the long-service cores at the material level than for direct comparison with engineering durability grades determined using standard full-size specimens.

### 3.2. Hydration Products and Phase Stability

#### 3.2.1. XRD Analysis

**Figure 7:**
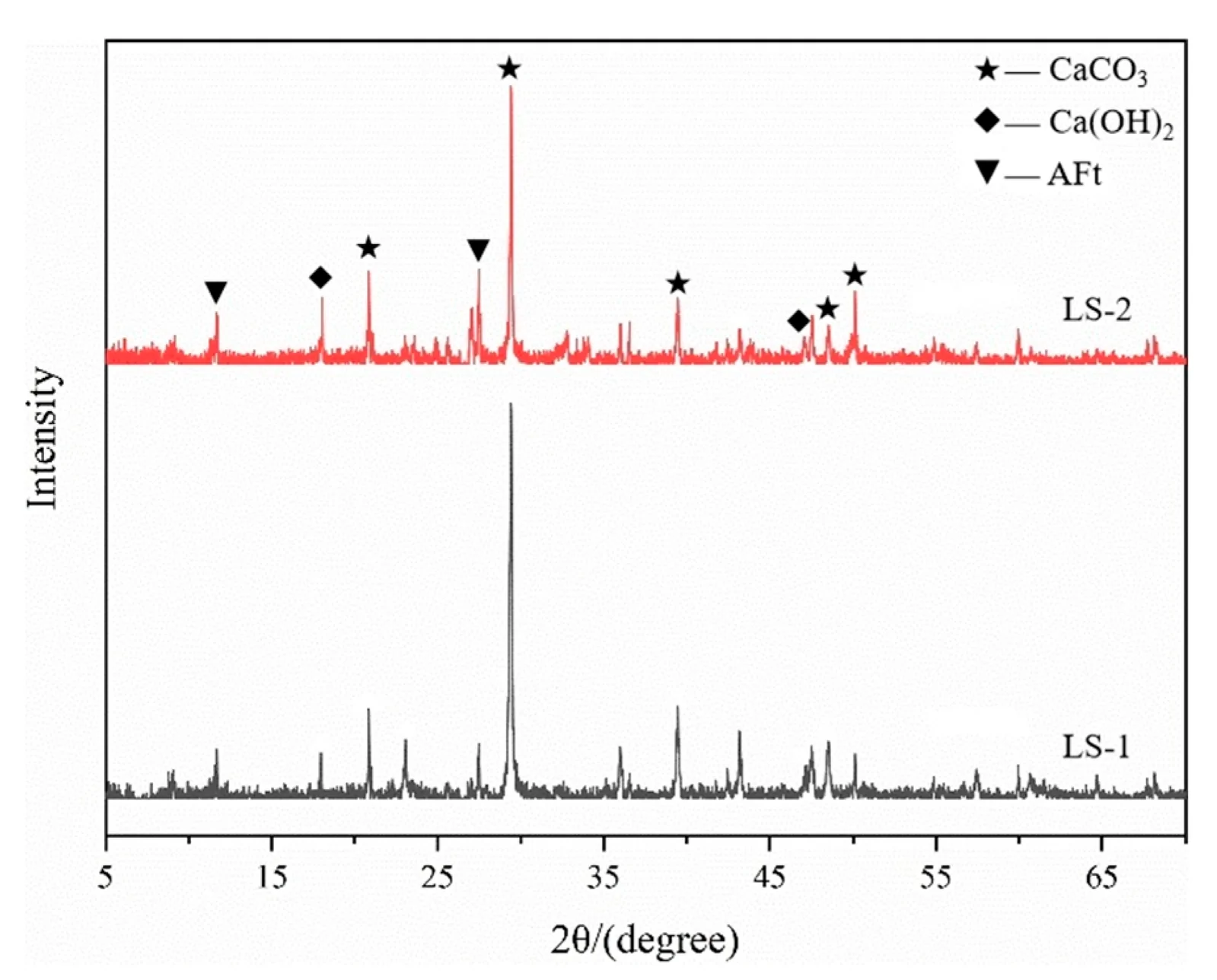
XRD patterns of the binder extracted from the dam concrete cores.

The XRD patterns of the binder extracted from the dam concrete cores are shown in Figure 7. The identifiable crystalline phases in the aged binder mainly included CaCO_3_, Ca(OH)_2_, and AFt. Among these phases, Ca(OH)_2_ and AFt are typical hydration products in cement-based systems, whereas C-S-H is generally difficult to detect directly by XRD because it is mainly present in an amorphous or poorly crystalline state. The relatively strong diffraction peaks of CaCO_3_ indicate that a certain degree of carbonation had occurred in the samples.

The XRD results indicate that the aged binder in the Lushui Dam still retained a relatively complete assemblage of major hydration products after more than 60 years of service, without showing obvious signs of phase instability or severe chemical deterioration. It should be noted that the strong CaCO_3_ peaks were more likely associated with long-term exposure of the extracted cores to air during storage, rather than directly indicating severe carbonation inside the dam during service. Despite the presence of carbonation, typical hydration products such as Ca(OH)_2_ could still be clearly identified, suggesting that the binder system of the long-service cores remained relatively stable at the phase level. This also provides phase-level support for the relatively good compressive strength and chloride ion penetration resistance observed in the cores.

#### 3.2.2. TG Analysis

**Figure 8:**
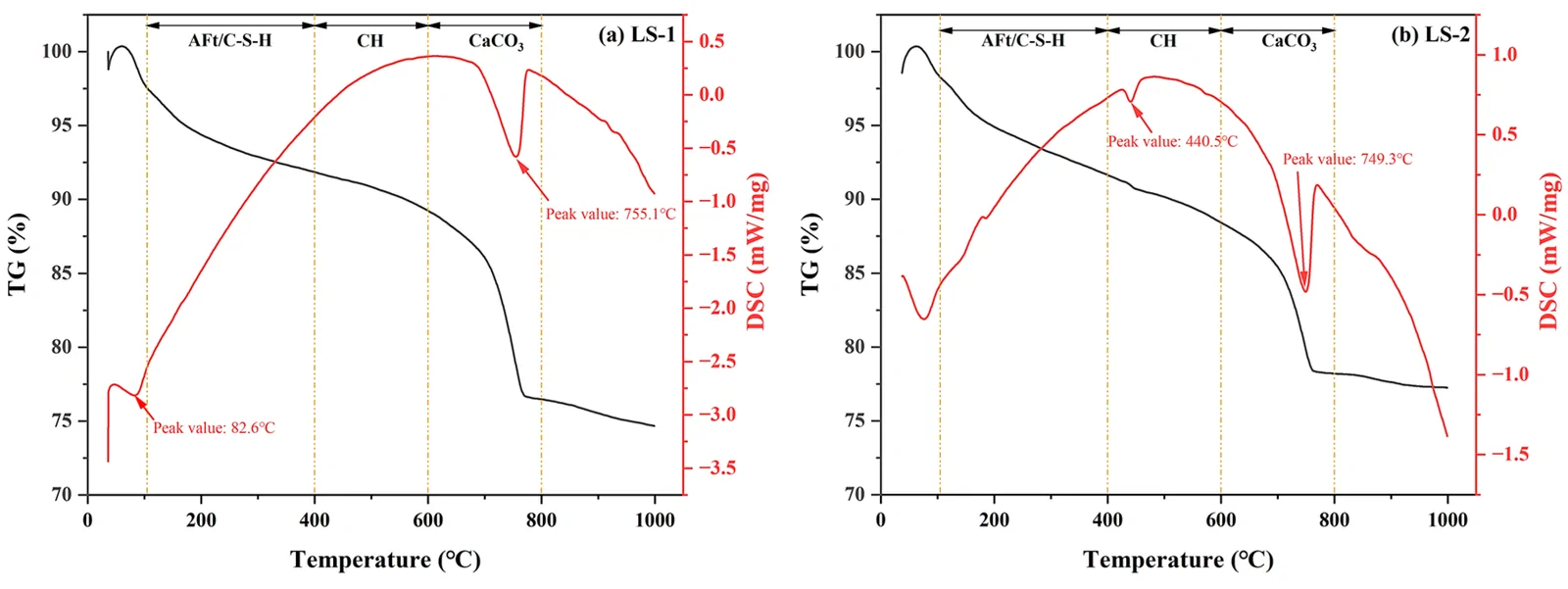
TG curves of the binder extracted from the dam concrete cores.

The TG curves of the binder extracted from the dam concrete cores are shown in Figure 8. The mass loss in the range of 105–400 °C mainly corresponds to the dehydration of C-S-H (together with a minor amount of AFt), the mass loss in the range of 400–600 °C corresponds to the decomposition of Ca(OH)_2_, and the mass loss in the range of 600–800 °C corresponds to the decomposition of CaCO_3_. These results indicate that the aged binder still contained major phase constituents, including C-S-H, AFt, Ca(OH)_2_, and CaCO_3_. In addition, the average mass losses of samples LS-1 and LS-2 in the above three ranges were 6.2 wt.%, 2.9 wt.%, and 11.5 wt.%, respectively. The content of C-S-H (together with AFt) was characterized by the mass loss of approximately 6.2 wt.% in the range of 105–400 °C; considering the variable composition of C-S-H, no fixed conversion factor was applied. According to Equations (5) and (6), the contents of Ca(OH)_2_ and CaCO_3_ were calculated to be 11.9 wt.% and 26.2 wt.%, respectively.

(5) $$m_{{\mathrm{Ca}(\mathrm{OH})}_{2}}={\Delta m}_{400-600{}^{\circ}C}\times \frac{M_{{\mathrm{Ca}(\mathrm{OH})}_{2}}}{M_{H_{2}O}}$$

(6) $$m_{{\mathrm{CaCO}}_{3}}={\Delta m}_{600-800{}^{\circ}C}\times \frac{M_{{\mathrm{CaCO}}_{3}}}{M_{{\mathrm{CO}}_{2}}}$$

where Δm is the corresponding mass loss and *M* is the molar mass.

The TG results further confirm the conclusions derived from the XRD analysis, indicating that the aged binder in the Lushui Dam still retained a relatively complete assemblage of major hydration products after more than 60 years of service. Compared with XRD, TG provides supplementary information on amorphous or poorly crystalline phases such as C-S-H and therefore better reflects the overall phase assemblage of the binder system. Based on the decomposition intervals of Ca(OH)_2_ and CaCO_3_, it can be inferred that the core samples, in their current state, still preserved typical hydration products while also being affected by a certain degree of carbonation. However, no obvious abnormal decomposition behavior or severe phase instability was observed, suggesting that the binder system remained relatively stable after long-term service.

Taken together, the XRD and TG results indicate that the aged binder of the Lushui Dam still retained a relatively complete hydration product system after more than 60 years of service. XRD identified the major crystalline phases, including CaCO_3_, Ca(OH)_2_, and Aft, while TG further verified the presence of C-S-H, Aft, Ca(OH)_2_, and CaCO_3_ from the perspective of thermal decomposition behavior. The good consistency between the two methods suggests that the basic chemical skeleton of the long-service binder system had not undergone significant deterioration. Although carbonation was present to some extent, the major hydration products were still identifiable, and no obvious abnormal decomposition behavior or pronounced phase instability was detected. This indicates that the dam cores remained in a relatively stable long-term hydration state after more than 60 years of service. In light of the macroscopic results presented above, the relatively complete hydration product system provides phase-level support for the favorable compressive strength and chloride ion penetration resistance of the cores, and also constitutes an important basis for the subsequent discussion of residual hydration capacity.

From the perspective of chemical reactions, the formation of the observed hydration products can be expressed as follows [Equations (7)–(10)]:

2C_3_S + 7H → C_3_S_2_H_4_ + 3CH (7)

2C_2_S + 5H → C_3_S_2_H_4_ + CH (8)

(9) $$C_{3}A+3C\bar{S}H_{2}+26H\to C_{6}A{\bar{S}}_{3}H_{32}$$

(10) $$C_{6}A{\bar{S}}_{3}H_{32}+2C_{3}A+4H\to 3C_{4}A\bar{S}H_{12}$$

where C = CaO, S = SiO_2_, A = Al_2_O_3_, $\bar{S}$ = SO_3_, H = H_2_O, and C$\bar{S}$H_2_ denotes gypsum. Equations (7) and (8) describe the formation of C-S-H gel and Ca(OH)_2_, and Equations (9) and (10) describe the formation of Aft and its conversion to Afm, which are consistent with the phases identified by XRD and TG.

Meanwhile, the CaCO_3_ detected in the aged binder mainly originates from carbonation, which can be described by the following reactions:

*Ca*(*OH*)_2_ + CO_2_ → CaCO_3_ + H_2_O (11)

C − S − H + CO_2_ → CaCO_3_ + SiO_2_ ⋅ nH_2_O (12)

Equation (11) corresponds to the direct carbonation of Ca(OH)_2_, and Equation (12) describes the carbonation of C-S-H; both contribute to the CaCO_3_ content quantified by TG. The good consistency among the XRD, TG, and chemical reaction analyses further confirms that the long-service binder retained a relatively complete and chemically stable hydration product system.

### 3.3. Microstructural Characteristics

#### 3.3.1. Morphology of the Binder

**Figure 9:**
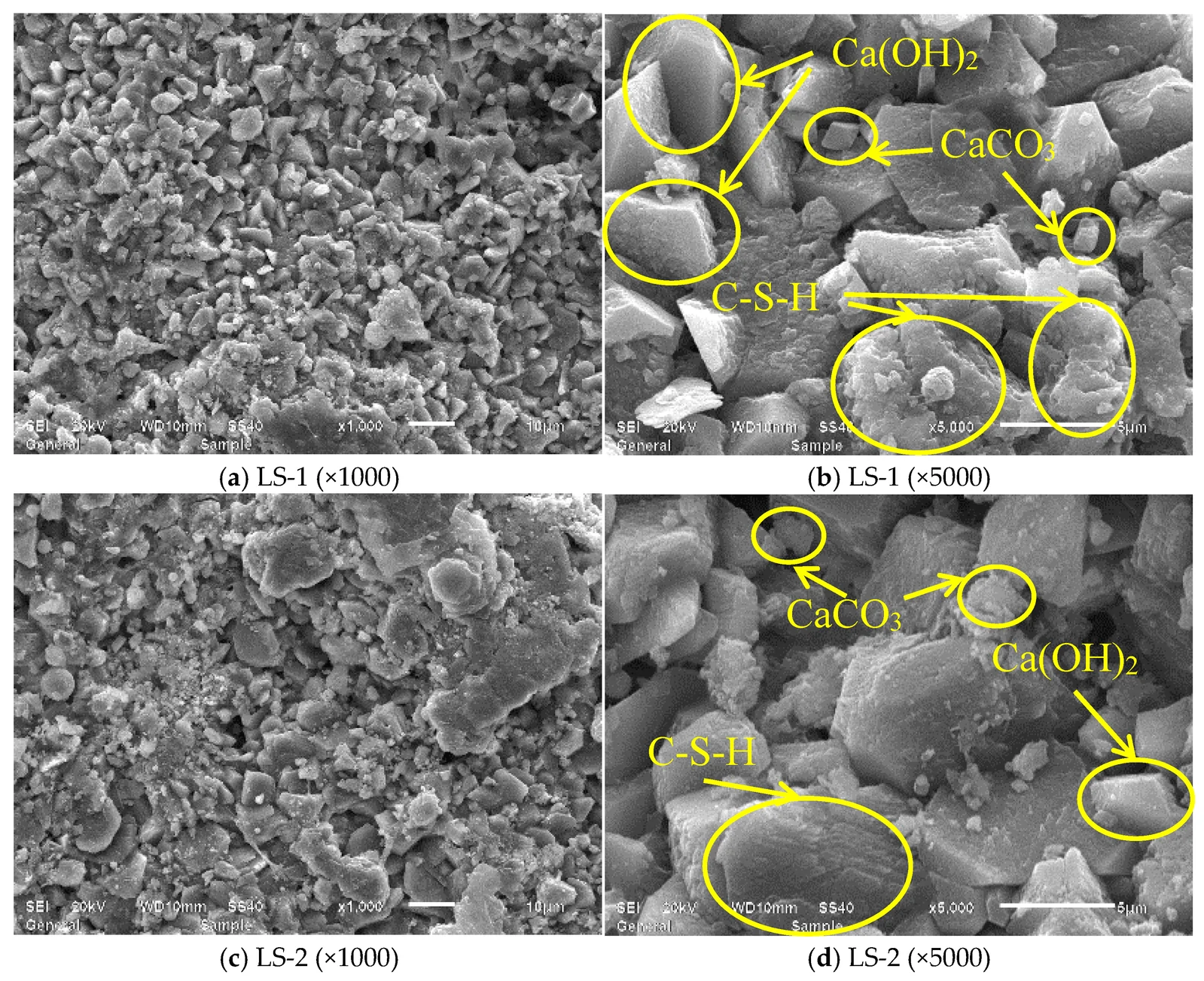
SEM images of the binder extracted from the dam concrete cores.

SEM images of the binder extracted from the dam concrete cores are shown in Figure 9. As highlighted in Figure 9, a large amount of layered Ca(OH)_2_ and irregularly shaped C-S-H gel can be observed, and these phases are interlaced to form a relatively compact local microstructure. Cubic CaCO_3_ crystals are also visible in the images. Needle-like Aft was not clearly observed, probably because it was covered by Ca(OH)_2_ and C-S-H gel.

The SEM observations indicate that, after more than 60 years of service, the binder of the Lushui Dam still retained a relatively intact local skeleton, without obvious large-scale loosening or severe deterioration. This is consistent with the XRD and TG results, which showed that the major hydration product system remained relatively complete, and it also provides microstructural support for the relatively good compressive performance of the cores. It should be noted that SEM observations only reflect the typical morphology of local regions. Therefore, they are more suitable for characterizing the general microstructural features of the long-service cores than for implying that the internal state of all samples was completely identical.

#### 3.3.2. Aggregate–Paste Interfacial Characteristics

**Figure 10:**
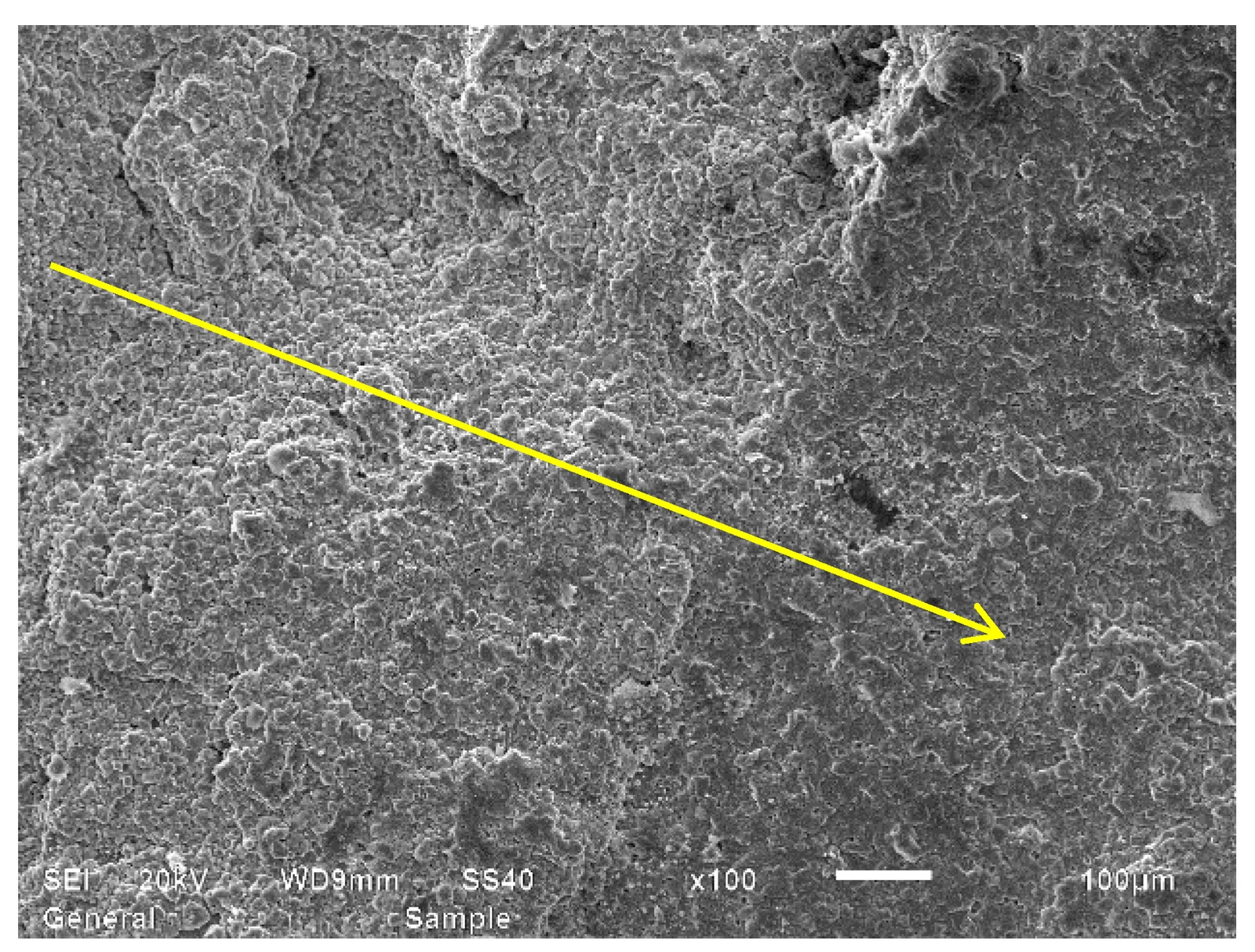
SEM images of the aggregate–paste interface in the dam concrete. (The arrow indicates the direction of the EDS scan).

**Figure 11:**
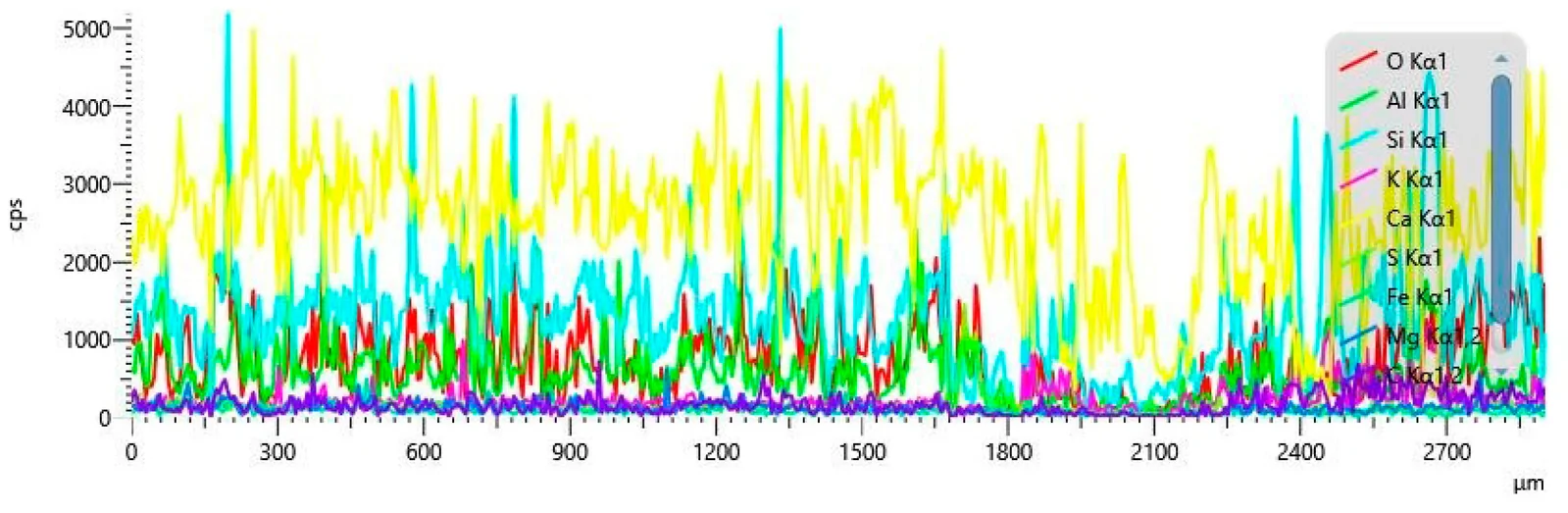
EDS spectra of the aggregate–paste interface in the dam concrete.

SEM images and EDS spectra of the aggregate–paste interface in the dam concrete are shown in Figure 10 and Figure 11, respectively. As shown in Figure 10, after long-term service, hydration products remained attached to and bonded with the aggregate surface. At this magnification, the field of view is dominated by the C-S-H-based paste matrix, and no obvious large-scale debonding or severe loosening was observed in the interfacial region. It should be noted that the aggregate–paste interface is irregular in reality; the position and thickness of the ITZ were identified on the basis of the EDS elemental distribution profile (Figure 11), in which a clear variation in the Ca and Si distributions was observed over the range of approximately 2070–2130 μm.

These results indicate that the aggregate–paste interfacial transition zone in the long-service cores of the Lushui Dam remained relatively stable after more than 60 years of service, without obvious signs of interfacial deterioration or instability. In concrete materials, the interfacial transition zone is generally regarded as a relatively weak region, and its condition has an important influence on mechanical performance and durability. The relatively continuous interfacial bonding observed in this study is consistent with the relatively good compressive strength and stable splitting tensile strength of the cores, suggesting that the aggregate and paste still maintained effective cooperative load transfer. It should be noted that the SEM/EDS results mainly reflect the interfacial state of the observed representative regions and are therefore more suitable for characterizing the general interfacial features of the long-service cores than for implying that the interfacial condition of all samples was completely identical.

#### 3.3.3. Pore Structure Characteristics

**Figure 12:**
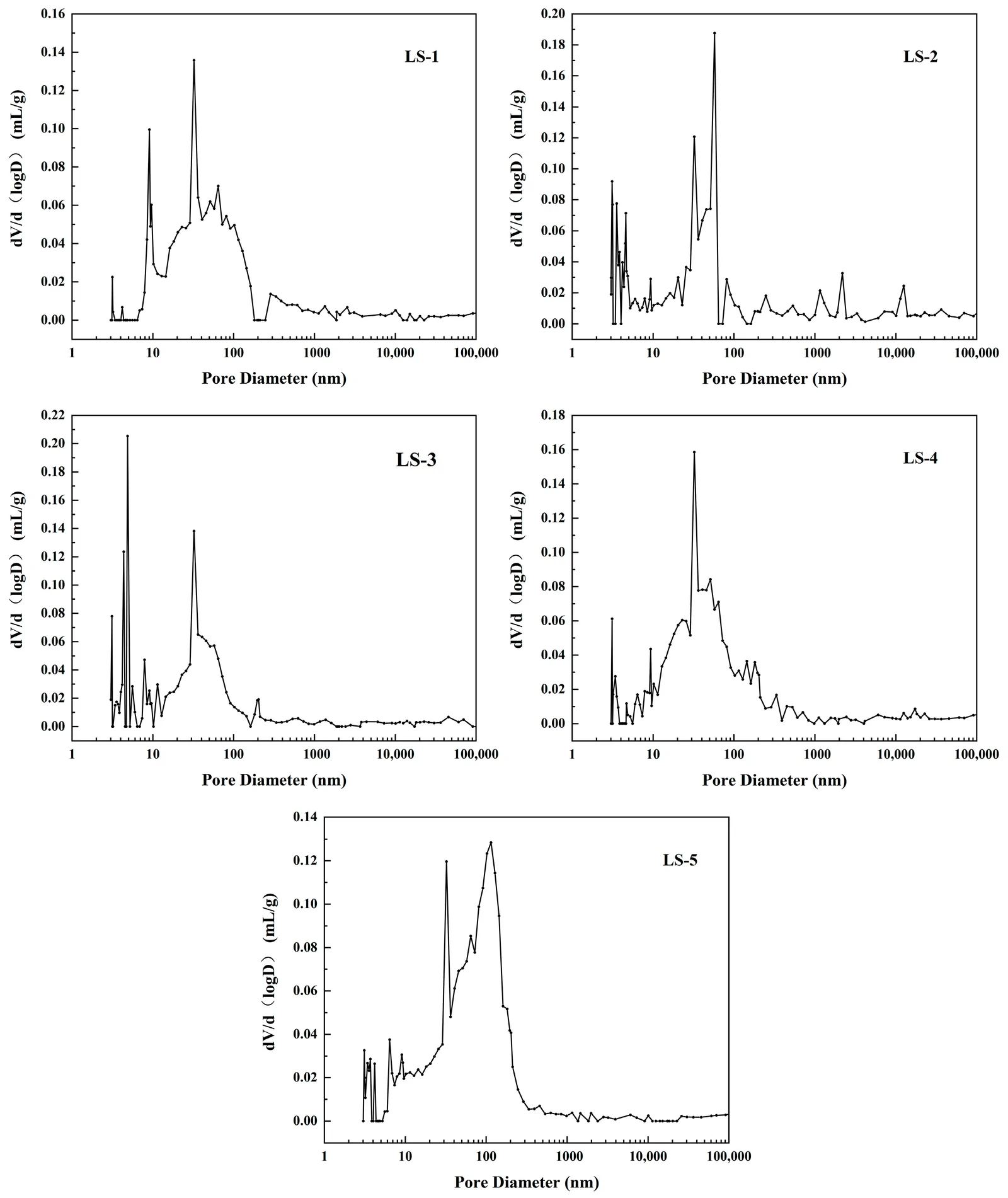
Pore size distribution curves of the binder extracted from the dam concrete.

The pore size distribution curves of the binder extracted from the dam concrete are shown in Figure 12, and the main pore structure parameters are summarized in Table 7. The pore volume of the different samples ranged from 5.95 × 10^−2^ to 20.06 × 10^−2^ cm^3^/g, the average pore diameter ranged from 15.9 to 79.3 nm, and the most probable pore diameter ranged from 4.85 to 114.43 nm, indicating obvious differences among the samples. According to the pore-size classification, two of the five samples were dominated by harmless pores smaller than 50 nm, whereas the other three were dominated by larger pores exceeding 100 nm. However, in some samples, the combined proportion of harmless and less harmful pores approached or exceeded 50%, indicating that the pore structure of the long-service cores was not uniform, but instead included both relatively dense samples and samples with a high proportion of larger pores.

The MIP results indicate that the long-service cores from the Lushui Dam exhibited a certain degree of spatial heterogeneity in pore structure, which is consistent with the sample-to-sample variability observed in the macroscopic mechanical properties and elastic modulus. In general, a higher proportion of harmless and less harmful pores helps reduce pore connectivity and is therefore favorable for maintaining good impermeability, whereas larger pores and locally unfavorable pore-size distributions are more likely to act as sensitive sites for freeze–thaw damage and local stress concentration. Therefore, the relatively dense pore structure observed in some samples is consistent with the generally favorable resistance to chloride ion penetration, while the pronounced pore-structure heterogeneity and relatively high proportion of large pores in some samples may be important mesoscopic reasons for the relatively poor frost resistance. It should be noted that mercury intrusion porosimetry reflects the pore structure characteristics of the selected hardened binder samples. Thus, the results are more suitable for describing the overall pore-structure trend of the long-service cores than for being directly extrapolated as the unique pore-structure state of the entire dam body.

Taken together, the SEM, SEM/EDS, and MIP results indicate that the long-service cores from the Lushui Dam exhibited an overall microstructural feature characterized by a relatively intact local binder skeleton, a relatively stable aggregate–paste interface, and a certain degree of pore-structure heterogeneity. The compact binder skeleton and stable interfacial transition zone are beneficial for maintaining the continuity of internal load-transfer paths, thereby providing a microstructural basis for the relatively good compressive strength and stable splitting tensile strength of the cores. The relatively refined and compact pore structure in some samples is also favorable for reducing the connectivity of internal transport pathways, which is consistent with the generally good resistance to chloride ion penetration. On the other hand, the heterogeneity of the pore structure and the relatively high proportion of larger pores in some samples suggest that local weak regions still existed inside the long-service cores, which may be an important microstructural reason for their relatively poor frost resistance. Overall, the long-service cores from the Lushui Dam exhibited a microstructural state of “overall stability with local differences,” providing an important basis for the subsequent discussion of residual hydration capacity and long-term service condition.

### 3.4. Residual Hydration Capacity of the Long-Service Binder

#### 3.4.1. Evaluation Based on Hydration Heat

**Figure 13:**
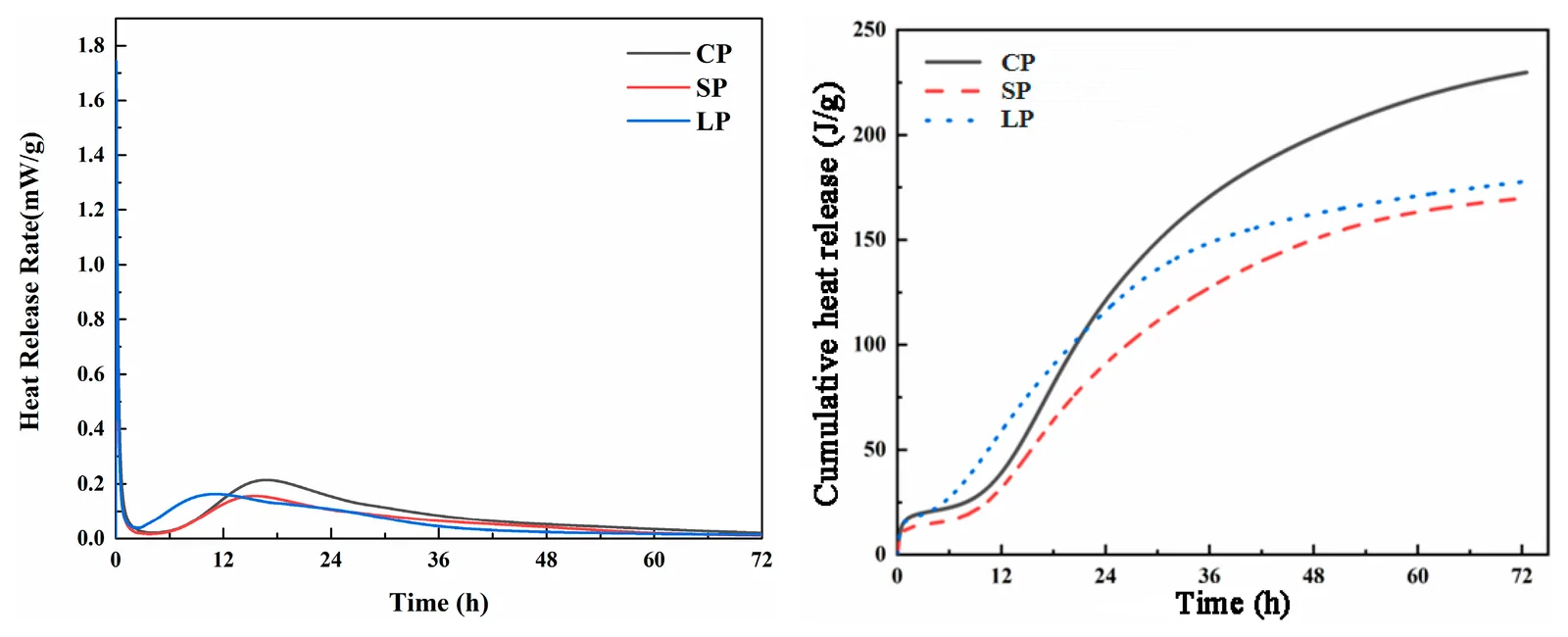
Hydration heat curves of the paste systems.

The hydration heat curves of the different paste systems are shown in Figure 13. As indicated by the heat flow curves, the LP system exhibited a relatively high initial dissolution peak, a significantly shorter induction period than the CP and SP systems, and an earlier onset and shorter duration of the acceleration period. The peak heat flow of the LP system was intermediate between those of the CP and SP systems. According to the cumulative heat release within 72 h, the cumulative heat values of the CP, LP, and SP systems were 230, 178, and 169 J/g, respectively, following the order CP > LP > SP. Based on Equation (1), the residual hydration capacity of the aged binder was calculated to be 14.75%.

These results indicate that the ground aged binder was not a completely inert component in the replacement system. The relatively high initial dissolution peak and shortened induction period of the LP system suggest that, after being introduced into the fresh system, the aged binder particles could accelerate early ion dissolution and promote the nucleation and growth of hydration products from the fresh cement. Such acceleration is consistent with the well-documented seeding effect of hydration products, whereby dispersed C-S-H particles provide additional nucleation sites and accelerate early-age hydration [40], as well as with the filler effect of finely divided particles [41]. Moreover, the residual unhydrated clinker within the aged binder may continue to hydrate upon re-exposure to water, further contributing to the observed early hydration activity [42]. In addition, the significantly higher cumulative heat release of the LP system compared with that of the SP system further indicates that its role was not limited to a simple dilution or filling effect, but involved a certain degree of overall activity.

It should be noted that the “residual hydration capacity” derived from the hydration heat results in this study is not a direct measure of the amount of unhydrated clinker, but rather a relative index based on the difference in thermal response among the replacement systems. This value may include not only the direct contribution of components in the aged binder that still retain reaction potential, but also combined effects such as ion release, nucleation promotion, and fine-particle filling. Nevertheless, the value of 14.75% indicates that the aged binder in the Lushui Dam had not become completely inert after more than 60 years of service, but still retained measurable relative activity in the fresh system. This also provides a basis for further evaluating its structural contribution through mortar strength tests.

#### 3.4.2. Evaluation Based on Mortar Strength

**Figure 14:**
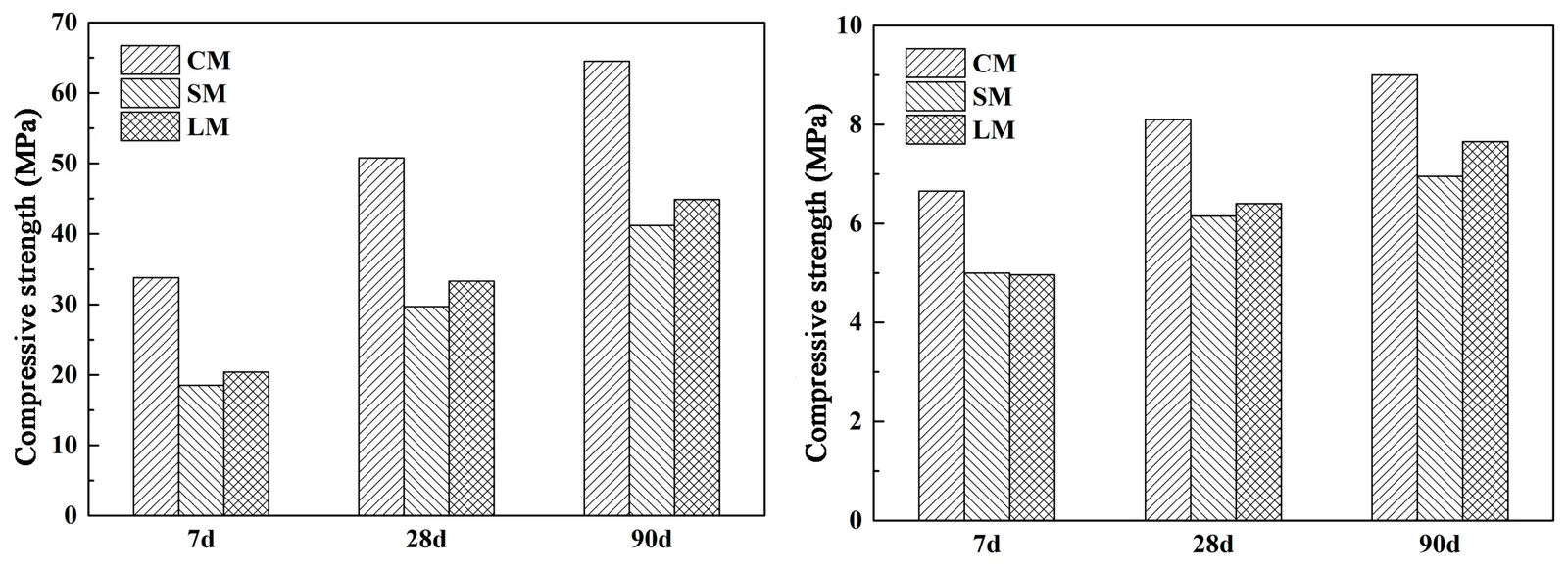
Compressive and flexural strengths of the mortar systems.

The compressive and flexural strength results of the different mortar systems are shown in Figure 14. The pure cement mortar system (CM) exhibited the highest strength, with compressive strengths of 50.8 and 64.5 Mpa at 28 and 90 d, respectively. The strengths of the system containing 30% ground quartz powder (SM) decreased significantly to 29.7 and 41.2 Mpa, respectively, whereas those of the system containing 30% ground aged binder (LM) were intermediate between the two, reaching 33.3 and 44.9 Mpa at 28 and 90 d, respectively. At the same curing age, the compressive strength of the LM system was consistently higher than that of the SM system. According to Equation (2), the residual hydration capacities of the aged binder calculated from the compressive strengths at 28 and 90 d were 17.06% and 15.88%, respectively. In addition, the flexural strength results followed a similar trend, namely CM > LM ≈ SM, indicating that the ground aged binder also played a positive role in later-stage mechanical development.

The mortar strength results further demonstrate that the role of the ground aged binder in the replacement system was not equivalent to that of an inert filler. If it had acted only as a filling material, the strength of the LM system would have been close to that of the SM system. However, the LM system was consistently stronger than the SM system at both 28 and 90 d, indicating that the aged binder made a measurable overall contribution to later-stage structural formation. This contribution may arise not only from components in the aged binder that still retain reaction potential, but also from nucleation promotion and fine-particle filling effects associated with the hydration products already present on the particle surfaces. Notably, the residual hydration capacities obtained from mortar strength, ranging from 15.88% to 17.06%, were close to the value of 14.75% obtained from the hydration heat method. This indicates that the residual activity of the aged binder was reflected not only in the early thermal response but also in the later mechanical behavior, thereby further supporting the conclusion that the binder system still retained a certain degree of reaction potential after more than 60 years of service.

Taken together, the hydration heat and mortar strength results show that the ground aged binder from the Lushui Dam consistently performed better than inert ground quartz powder in the replacement systems. The residual hydration capacity calculated from the 72 h cumulative heat release was 14.75%, whereas those calculated from the compressive strengths at 28 and 90 d were 17.06% and 15.88%, respectively. These values were close to each other and all fell within the range of 14–17%, indicating that the activity of the aged binder was not accidental but could be detected in both the early reaction process and later mechanical development. It should be emphasized that the “residual hydration capacity” defined in this study is more appropriately interpreted as an overall activity index of the aged binder in fresh systems, rather than as evidence that the binder retained the same proportion of its original cement activity. In combination with the XRD, TG, and microstructural results presented above, it can be inferred that the binder system in the long-service cores from the Lushui Dam had not become completely inert after more than 60 years of service, but still retained a certain degree of residual reactivity. This finding contributes to a deeper understanding of the material state of long-service hydraulic concrete, suggesting that the sustained performance of such materials may be related not only to the relative integrity of the existing structure, but also to the fact that the potential activity of the binder system had not been completely exhausted.

### 3.5. Discussion

Taken together, the results of the macroscopic property tests, phase composition analysis, microstructural characterization, and residual hydration capacity evaluation indicate that the precast concrete blocks of the Lushui Dam remained in a relatively stable material state after more than 60 years of service. On the one hand, the compressive strength of the cores was mainly distributed within the range of 20–40 MPa, the splitting tensile strength was mainly distributed within the range of 1–3 MPa, and the resistance to chloride ion penetration was generally favorable. On the other hand, the XRD, TG, SEM, and MIP results showed that the binder still retained a relatively complete assemblage of major hydration products. Meanwhile, the local binder skeleton and aggregate–paste interface remained relatively stable, and some samples still exhibited relatively dense pore structure characteristics. These results collectively indicate that the long-service cores did not show obvious overall deterioration or instability, but still maintained a relatively complete phase assemblage and structural basis.

At the same time, different performance indicators of the long-service cores did not necessarily evolve synchronously. Compared with the relatively good compressive strength and chloride ion penetration resistance, the frost resistance was markedly weaker, suggesting a certain degree of performance divergence in long-service dam concrete. From a mesostructural perspective, the relatively complete hydration product system, stable binder skeleton, and relatively intact interfacial condition provided the material basis for the preservation of load-bearing capacity and impermeability. In contrast, the heterogeneity of the pore structure and the relatively high proportion of large pores in some samples may be important reasons for the higher sensitivity of the material to freeze–thaw action. In addition, the aged binder still exhibited a residual hydration capacity of approximately 14–17%, indicating that the binder system in the dam had not reached a completely inert end state but still retained a certain degree of residual reaction potential under suitable conditions. This finding suggests that the sustained performance of long-service dam concrete may be related not only to the current structural integrity of the material, but also to the fact that the potential activity of the binder system had not been completely exhausted.

It should be emphasized that the present results mainly reflect the long-term material state of the precast concrete blocks in Monolith No. 3 of the main dam, and that some durability-related tests were constrained by core size and specimen conditions. In addition, the phase-analysis results may also have been affected by carbonation induced during long-term exposure of the extracted cores to air during storage. Therefore, the conclusions of this study are more appropriate for identifying the material state of long-service dam concrete at the material level than for direct extrapolation to a quantitative evaluation of the overall service performance of the entire dam. Nevertheless, by systematically characterizing the material state of dam concrete after more than 60 years of service from the perspectives of macroscopic performance, phase composition, microstructure, and residual hydration capacity, this study provides a useful reference for the condition identification and maintenance evaluation of existing dam concrete.

Durability implications and maintenance recommendations. The results indicate that frost resistance, rather than chloride ion penetration resistance, is the controlling durability indicator for the long-service dam concrete in the Lushui region. The failure of the frost resistance specimens within 25–50 freeze–thaw cycles suggests that the material has undergone cumulative mesoscopic damage under cyclic frost action. For engineering practice, a phased maintenance strategy is recommended: (i) in the short term, routine inspection and monitoring of the water-level fluctuation zone and exposed surfaces, with emphasis on crack development and surface scaling; (ii) in the mid term, surface protection (e.g., hydrophobic impregnation or protective coating), crack sealing, and improvement of local drainage to reduce water saturation during freezing; and (iii) in the long term, periodic re-assessment of the material condition and, if necessary, local repair or strengthening. A quantitative service-life prediction based on site-specific freeze–thaw exposure data and deterioration models will be conducted in future work.

## 4. Conclusions

In this study, concrete cores extracted from the Lushui Reservoir Dam after more than 60 years of service were investigated to systematically evaluate their long-term macroscopic properties, hydration product composition, microstructural characteristics, and the residual hydration capacity of the aged binder. Based on the experimental results and the above discussion, the following conclusions can be drawn:

(1) The precast concrete blocks of the Lushui Dam still retained relatively good basic mechanical properties after more than 60 years of service. The compressive strength of the cores was mainly distributed within the range of 20–40 MPa, and the splitting tensile strength was mainly distributed within the range of 1–3 MPa, indicating that no overall strength deterioration occurred in these long-service cores. In addition, the resistance to chloride ion penetration was generally favorable, whereas the frost resistance was relatively poor. This suggests that different macroscopic performance indicators of long-service dam concrete do not necessarily evolve synchronously, but may instead exhibit a certain degree of performance divergence.
(2) The XRD, TG, SEM, SEM/EDS, and MIP results showed that the aged binder still retained a relatively complete assemblage of major hydration products. Meanwhile, the local binder skeleton and aggregate–paste interface remained relatively stable, and some samples still exhibited relatively dense pore structure characteristics. These findings indicate that the dam cores still possessed a relatively stable phase assemblage and microstructural basis after more than 60 years of service, providing material-level support for their favorable compressive strength and chloride ion penetration resistance. Meanwhile, the heterogeneity of the pore structure and the relatively high proportion of large pores in some samples may be important mesoscopic reasons for their relatively poor frost resistance.
(3) The residual hydration capacity of the aged binder, calculated from hydration heat and mortar strength results, was approximately 14–17%, indicating that the binder in the dam had not become completely inert after more than 60 years of service, but still exhibited measurable overall activity in fresh systems. This result suggests that the sustained performance of long-service dam concrete may be related not only to the integrity of its existing structure, but also to the fact that the potential activity of the binder system had not been completely exhausted.

## Figures and Tables

**Table 1:** Main physical properties of the reference cement.

Cement	Specific Surface Area (m^2^/kg)	Density (g/cm^3^)	Water Demand for Standard Consistency (%)	Setting Time (Min)		Flexural Strength (MPa)		Compressive Strength (MPa)	
				Initial Setting	Final Setting	3 d	28 d	3 d	28 d
P.MH 42.5	350	3.18	24.8	215	355	5.7	8.5	25.7	50.8

**Table 2:** Main chemical composition of the reference cement (wt.%).

Component	CaO	SiO_2_	Al_2_O_3_	Fe_2_O_3_	MgO	SO_3_	LOI
Content	58.24	35.26	1.13	1.37	1.35	1.15	1.20

**Table 3:** Mix proportions of paste specimens for the hydration heat test.

Mix ID	Water-to-Binder Ratio	Cement	Ground Quartz Powder	Ground Binder Powder
CP	0.5	100%	/	/
SP	0.5	70%	30%	/
LP	0.5	70%	/	30%

**Table 4:** Mix proportions of mortar specimens for the strength test.

Mix ID	Water-to-Binder Ratio	Binder-to-Sand Ratio	Cement	Ground Quartz Powder	Ground Binder Powder
CM	0.5	1/3	100%	/	/
SM	0.5	1/3	70%	30%	/
LM	0.5	1/3	70%	/	30%

**Table 5:** Frost resistance test results of the dam concrete cores.

No.	Borehole No.	Core Segment ID	Specimen Size (mm)	Mass Loss During Freeze–Thaw Cycling (%)	
				25 Cycles	50 Cycles
1	SP1	3 (2/10)	Φ54.3 × 162.9	Failed	—
2		1 (1/2)	Φ54.3 × 162.9	Failed	—
3		2 (1/1)	Φ54.3 × 162.8	0.83	Failed
4	CZ1	1 (1/2)	Φ54.4 × 163.1	Failed	—
5		4 (2/9)	Φ54.4 × 163.2	Failed	—

**Table 6:** Chloride ion penetration resistance test results of the dam concrete cores.

No.	Borehole No.	Core Segment ID	Specimen Size (mm)	Electric Flux (C)	
				Measured Value	Converted Value
1	SP1	3 (2/10)	Φ54.3 × 50.2	1300	3980
2		1 (1/2)	Φ54.3 × 50.1	920	2820
3		2 (1/1)	Φ54.3 × 50.1	530	1620
4	CZ1	1 (1/2)	Φ54.4 × 50.2	1060	3240
5		4 (2/9)	Φ54.4 × 50.0	1020	3120

**Table 7:** Pore structure characteristics of the binder extracted from the dam concrete.

Sample	Porosity (%)	Pore Volume (10^−2^ cm^3^/g)	Average Pore Diameter (nm)	Most Probable Pore Diameter (nm)	Pore Size Distribution (%)		
					d ≤ 50 nm	50 nm < d < 100 nm	d ≥ 100 nm
LS-1	29.96	20.06	79.3	32.24	20.05	8.55	71.5
LS-2	15.96	8.79	23.7	57.35	42.48	17.58	39.94
LS-3	10.98	5.95	15.9	4.85	65.02	20.04	14.94
LS-4	22.34	14.13	46	32.26	33.5	12.31	54.19
LS-5	19.55	10.90	35.8	114.43	32.58	23.55	43.87

## Data Availability

The original contributions presented in this study are included in the article. Further inquiries can be directed to the corresponding author.
